# Aggressive behavior of anaplastic undifferentiated carcinoma arising from the hilar bile duct

**DOI:** 10.1186/s40792-022-01368-y

**Published:** 2022-01-17

**Authors:** Masayuki Akita, Eri Maeda, Ryo Ishida, Tatsuya Morikawa, Tohru Nishimura, Koichiro Abe, Akihito Kozuki, Tomohiro Tanaka, Yukihiro Imai, Kunihiko Kaneda

**Affiliations:** 1 Department of Surgery, Kakogawa Central City Hospital, Kakogawa, 675-8611 Japan; 2 Depertment of Diagnostic Pathology, Kakogawa Central City Hospital, Kakogawa, Japan

**Keywords:** Undifferentiated carcinoma, Hilar cholangiocarcinoma

## Abstract

**Background:**

Undifferentiated carcinoma of the biliary tree is extremely rare, and biliary undifferentiated carcinoma mostly originates from the gallbladder. We herein present a case of anaplastic undifferentiated carcinoma of the hilar bile duct and reviewed the literature.

**Case presentation:**

The patient was an 81-year-old male with obstructive jaundice. Contrast-enhanced computed tomography (CT) showed a protruded tumor located at the hepatic hilum. Obstructive jaundice was relieved by endoscopic drainage. Endoscopic biopsy revealed carcinoma without glandular differentiation, and the patient was diagnosed with resectable hilar undifferentiated carcinoma. During the 5-week preoperative examination, the tumor increased in size from 23 to 45 mm. Left hemi-hepatectomy and extrahepatic bile duct resection were performed, and there were no postoperative complications. Histological findings demonstrated that the tumor was mainly composed of non-cohesive polygonal neoplasms with pleomorphic nuclei, and was diagnosed as anaplastic undifferentiated carcinoma of the common hepatic duct (T2a N0 M0 Stage II). One month after surgery, the patient was readmitted to our hospital with pyrexia due to cholangitis, and liver nodules suggestive of multiple liver metastases were detected by CT. Three months after surgery, the patient died of multiple liver metastases.

**Conclusions:**

This is the first case report of undifferentiated cholangiocarcinoma with anaplastic features. Anaplastic undifferentiated carcinoma of the hilar bile duct showed preoperative rapid growth and early relapse despite a cancer-negative surgical margin.

## Background

Biliary malignancies are commonly adenocarcinoma with various degrees of ductular differentiation. Undifferentiated carcinoma is extremely rare and displays more aggressive behavior than differentiated tumors [1–4]. The majority of reported cases of undifferentiated carcinoma in the biliary tree have been gallbladder cancer.

Various nomenclatures are used for tumors with a sarcomatoid appearance: carcinosarcoma, so-called carcinosarcoma, sarcomatoid carcinoma, and undifferentiated carcinoma. Carcinosarcoma is frequently used for tumors with both adenocarcinoma and stromal element, such as osteoid, chondroid, or rhabdoid components. So-called carcinosarcoma commonly represents tumors comprising carcinomatous and sarcomatous cells. However, the differences among these terms have not been clearly defined, and, thus, they have sometimes been confused with each other.

We encountered a case of hilar anaplastic undifferentiated carcinoma. The tumor showed exponential expansion during a preoperative assessment. Despite a cancer-negative surgical margin, the patient died due to very early liver recurrence. We herein described this case and reviewed previous cases of hilar undifferentiated carcinoma.

## Case presentation

An 81-year-old male was admitted to our hospital with general malaise and brown urine. He had previous histories of hypertension, diabetes mellitus, and chronic renal failure. Laboratory blood examinations revealed elevated levels of serum aspartate aminotransferase (497 U/L, normal 13–16), alanine aminotransferase (703 U/L, normal 10–42), total bilirubin (15.3 mg/dl, normal 0.4–1.5), and carbohydrate antigen 19-9 (196 U/ml, normal 0–37).

**Fig. 1:**
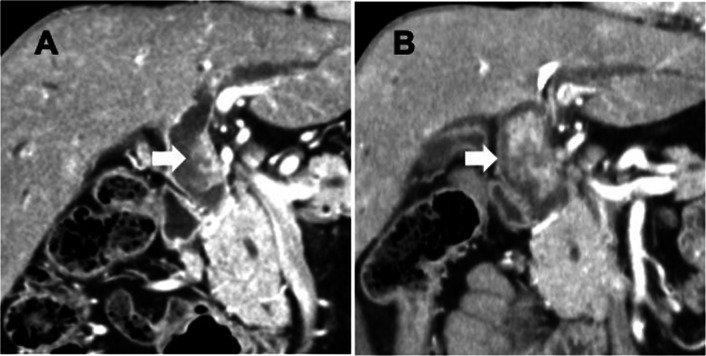
Computed tomography images at presentation showed a nodular mass at the common hepatic duct (**A**). Imaging before surgery showed rapid tumor growth (**B**)

**Fig. 2:**
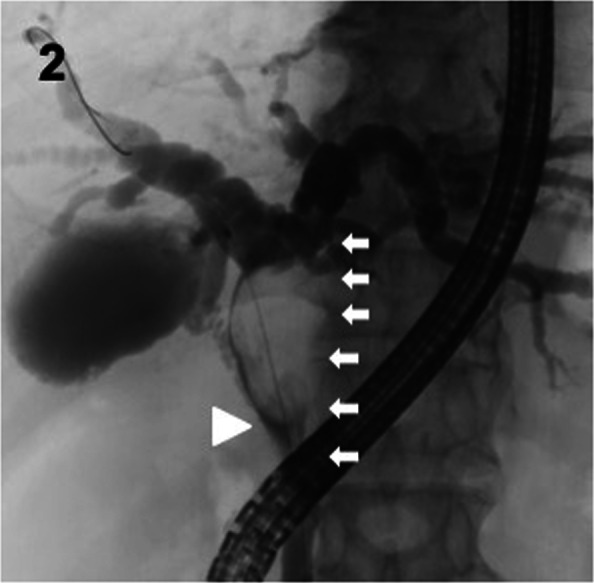
Endoscopic retrograde cholangiography revealed a protruding tumor in the hilar bile duct with intrahepatic bile duct dilatation. Repeated biopsies showed that the tumor extended from the hepatic duct confluence to the intrapancreatic bile duct around the superior edge of the pancreas. A white arrowhead shows the root of the cystic duct

Computed tomography (CT) and endoscopic retrograde cholangiography revealed a protruded tumor arising from the left wall of the common hepatic duct with dilation of the intrahepatic bile duct (Figs. 1A, 2). CT imaging also showed regional lymph node swelling in the hepatoduodenal ligament and supra-pancreatic area. Tumor invasion to the right hepatic artery was not identified, and distant metastasis was not detected by fluorodeoxyglucose–positron emission tomography. Endoscopic biliary drainage was performed to relieve jaundice and an endoscopic retrograde biliary drainage tube was placed into the left hepatic bile duct. Repeated endoscopic bile duct biopsies revealed malignant epithelial cells without glandular differentiation and also that the main tumor was located in the common hepatic duct. Mapping biopsy showed that the tumor extended from the hepatic duct confluence to the intrapancreatic bile duct around the superior edge of the pancreas (Bismuth type II).

The patient needed additional endoscopic biliary drainage due to the insufficient alleviation of jaundice. A preoperative tumor assessment and the amelioration of jaundice required 5 weeks, and the tumor increased in size from 23 to 45 mm during this period (Fig. 1B).

Before surgery, the indocyanine green retention rate at 15 min (ICG–15R) was 13.7% and ICG-K 0.124. The calculated remnant right liver volume was 55% by three-dimensional CT with Synapse Vincent (Fujifilm, Tokyo, JAPAN). We then planned (1) segmental bile duct resection (BDR), (2) left hemihepatectomy with extrahepatic bile resection, or (3) subtotal stomach-preserving pancreatoduodenectomy based on the intraoperative frozen section diagnosis of the bile duct stump because of the age of the patient.

During surgery, we initially collected samples of white liver nodules and para-aortic lymph nodes (station 16b1 int.), which were confirmed to be negative for malignancy in the intraoperative rapid diagnosis. After dissection of the lymph nodes in the hepatoduodenal ligament and superior- and inferior-pancreatic area, the intrapancreatic bile duct was resected 2 cm from the duodenal papilla. The distal duct stump was also negative for malignancy. We then resected the right and left hepatic bile ducts, and invasive cancer was intraoperatively confirmed in the left hepatic duct only. Left hemi-hepatectomy and caudate lobectomy with bile duct reconstruction were performed.

**Fig. 3:**
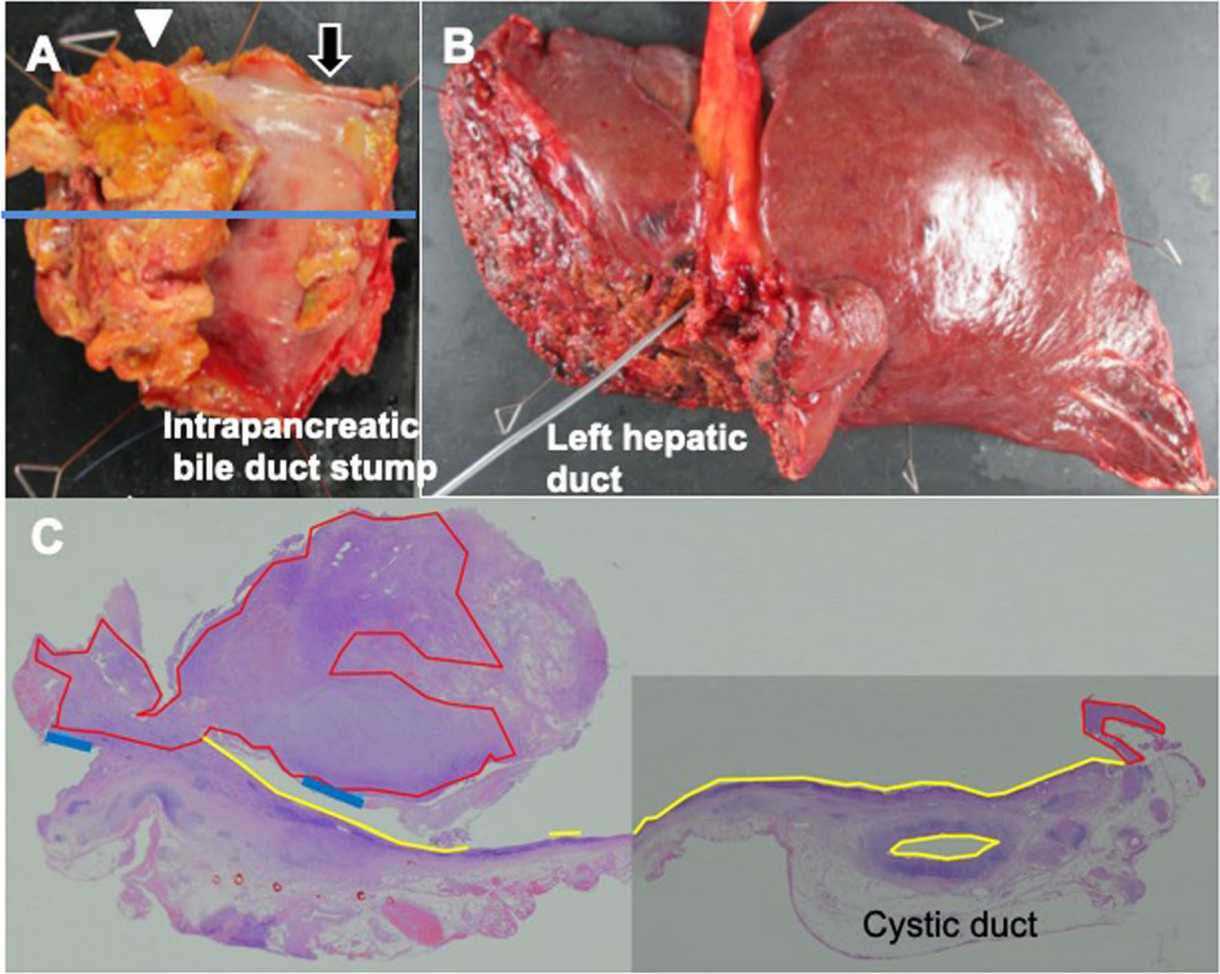
Resected specimen of the extrahepatic bile duct. The stumps of the intrapancreatic bile duct and right hepatic duct (black arrow) were negative for malignancy. Invasive cancer was observed at the stump of the left hepatic duct (white arrowhead). The blue line shows the slice of (**A**). Biliary intraepithelial neoplasm-3 was widely observed in the left intrahepatic bile duct (**B**). The protruded tumor mainly consisted of undifferentiated carcinoma (red) and a differentiated component located in the peripheral of the nodule (blue). High-grade biliary intraepithelial neoplasms (BilIN-3) were observed around the nodule (yellow) (**C**)

**Fig. 4:**
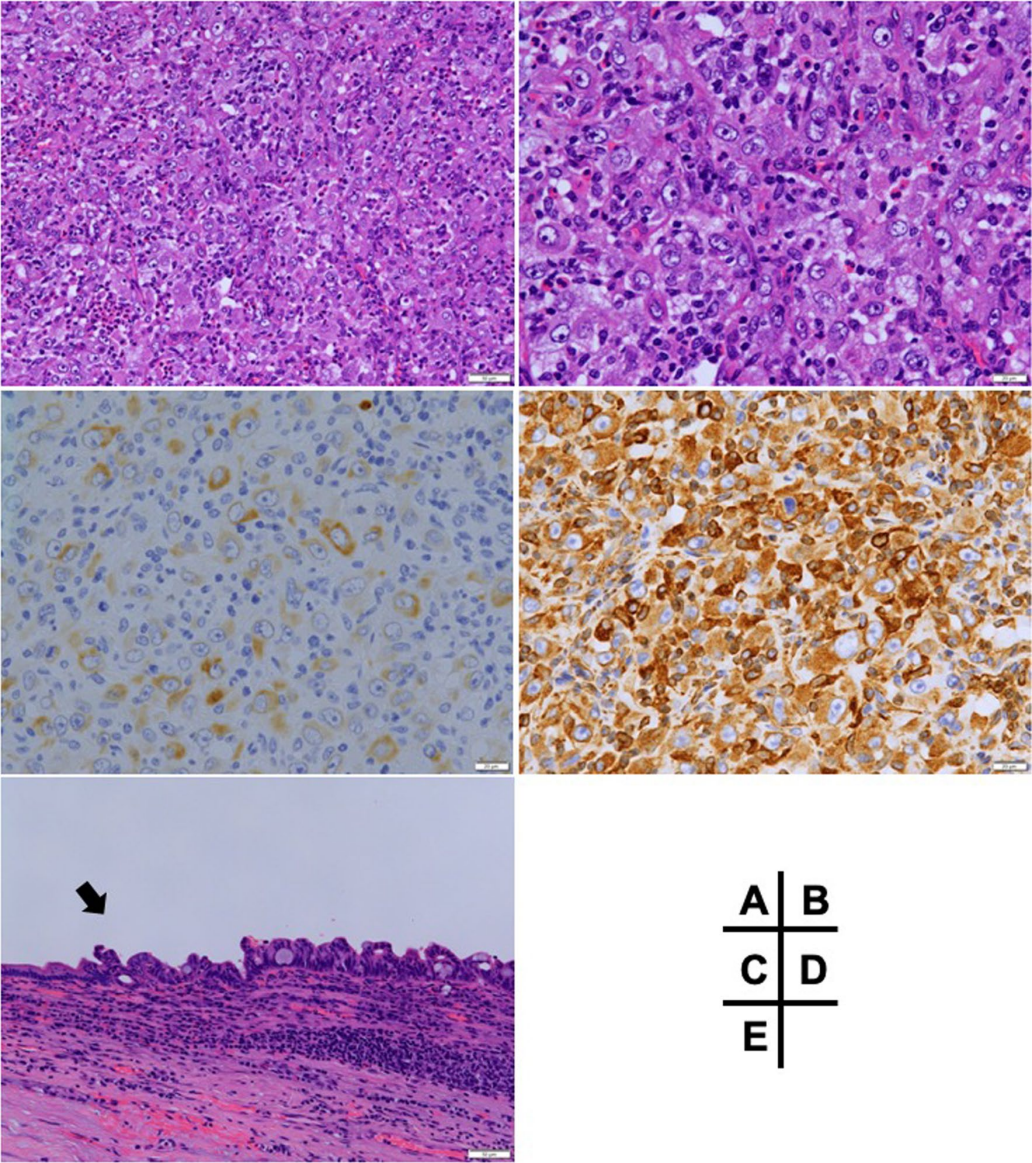
Tumor was mainly composed of pleomorphic atypical cells without ductal formation (**A** H/E × 200, **B** H/E × 400). Cytokeratin staining was weakly positive in these cells, while vimentin expression was strongly positive (**C** cytokeratin × 400, **D** vimentin × 400). **E** shows the transitional area from the normal biliary epithelium to a high-grade biliary intraepithelial neoplasm in the left intrahepatic duct (BilIN-3)

Pathological findings showed that the protruding nodular tumor (50 × 35 mm) was located from the common hepatic duct to the left intrahepatic bile duct (Fig. 3A, B). The tumor mainly contained pleomorphic mononuclear cells indicating undifferentiated carcinoma (more than 95% of the tumor, highlighted in red in Fig. 3C), and well-moderately differentiated adenocarcinoma was only observed around the periphery of the tumor (highlighted in blue in Fig. 3C). Biliary intraepithelial neoplasm-3 was noted from the left intrahepatic bile duct to the cystic duct (Bismuth type IIIb). There was lympho-vascular infiltration, but no lymph node metastasis. In an immunohistological analysis, the undifferentiated component was strongly positive for vimentin and weakly positive for CK AE1/AE3 (Fig. 4), but negative for Periodic Acid Schiff staining. The pathological diagnosis according to the WHO classification of tumors and the TNM staging system of UICC 8th edition was Stage II (T2a N0 M0) perihilar anaplastic undifferentiated carcinoma [5, 6].

The postoperative course was uneventful and the patient was discharged from hospital 23 days after surgery. One month after surgery, he was re-admitted to the hospital with cholangitis. CT imaging at that time showed liver nodules suggestive of multiple liver metastases. Due to his poor general condition, the patient was unable to receive chemotherapy for recurrence. Three months after surgery, he died of the disease.

## Discussion

Undifferentiated carcinoma originating from the biliary tree is extremely rare. Undifferentiated carcinoma does not have a definitive direction of differentiation, and is microscopically characterized by a non-glandular and non-descript morphology. Various subtypes have been reported, with some mainly having the spindle cell morphology, while others comprise pleomorphic mononuclear cells [7–9]. Carcinosarcoma also belongs to undifferentiated carcinoma and comprises both conventional ductal adenocarcinoma and sarcomatoid elements [10]. Sarcomatoid components sometimes show heterologous differentiation.

According to the World Health Organization classification (5^th^ edition), there is no histological definition for undifferentiated carcinoma in extrahepatic bile duct section [11]. On the other hand, by referring to pancreas sections as a counterpart of the bile duct, undifferentiated carcinomas are classified into three subtypes: anaplastic undifferentiated carcinoma, sarcomatoid undifferentiated carcinoma, and carcinosarcoma [5]. The anaplastic type is composed of at least 80% of neoplasms with pleomorphic nuclei, the sarcomatoid type is characterized by at least 80% of cells showing spindle cell features, and carcinosarcoma comprises both sarcomatoid and conventional adenocarcinoma components (each element constitutes more than 30% of the tumor). In the present case, carcinoma was mainly composed of pleomorphic mononuclear cells and differentiated adenocarcinoma was only located around the periphery of the tumor. We hypothesized that undifferentiated components came from the de-differentiation of that adenocarcinoma.

Six cases of perihilar undifferentiated carcinoma, including the present case, have been reported to date (Table 1) [12–16]. We searched for these cases using MEDLINE with following keywords: “undifferentiated carcinoma, sarcomatoid carcinoma, carcinosarcoma, hilar bile duct, hepatic hilum”. We retrospectively reviewed their pathological findings, and presumptively classified them based on the criteria described above. Most cases had a sarcomatoid component featured by spindle shape cells (4 sarcomatoid types and one carcinosarcoma), while one had a cartilage component. In-hospital mortality was reported for two sarcomatoid cases (liver failure and pulmonary infarction), and early tumor relapse was noted in one sarcomatoid and anaplastic cancer each (2 and 1 months after surgery, respectively). A previous review of biliary carcinosarcoma showed that patients with Stage II or more advanced extrahepatic tumors did not survive more than 3 years after surgery, and another study reported the more aggressive malignant behavior of undifferentiated carcinoma than differentiated tumors [1, 4].

**Table 1:** Patient characteristics with undifferentiated carcinoma of the hepatic hilus

Author	Sex/Age	Chief complaint	Gross appearance	Bismuth	Size (mm)	Surgical procedure	Resection status	Subtype	Prognosis (the cause of death)
Yuan [12]	62/M	Jaundice	Nodular	IV	35 × 20	Left hepatectomy + extrahepatic BDR	-	Sarcomatoid	POD10 dead (liver failure)
Sodergren [13]	64/F	Appetite loss	Polypoid	II	20 × 12	BDR	-	Carcinosarcoma	5 years alive
Nakanishi [14]	59/M	Jaundice	Nodular	IV	40 × 20	Right hepatectomy + extrahepatic BDR	-	Sarcomatoid	POD11 dead (Pulmonary infarction)
Ide [15]	67/M	Jaundice	Nodular	II	17 × 12	BDR	R0	Sarcomatoid	16 months alive
Lee [16]	91/F	–	Nodular	II	–	BDR	–	Sarcomatoid	2 months dead (−)
Present case	80/M	Jaundice rown urine	Nodular	IIIb	50 × 35	Left hepatectomy + extrahepatic BDR	R0	Anaplastic	3 months dead (liver metstasis)

*BDR* bile duct resection

In the present study, we applied WHO classification for subclassifying perihilar undifferentiated carcinoma. However, according to AFIP ATLAS OF TUMOR PATJOLOGY, which is another widely accepted atlas of pathology, undifferentiated carcinoma in the extrahepatic bile duct is characterized by spindle and giant malignant cells with a minor component of differentiated adenocarcinoma [17]. We think that one of the major problems to resolved in undifferentiated carcinoma is how to diagnose and classify the disease, and the diagnostic criteria of undifferentiated carcinoma vary among the referring books. Furthermore, AFIP ATLAS distinguishes extrahepatic carcinosarcoma from undifferentiated carcinoma. While whether sarcmatoid and anaplastic subtypes, and carcinosarcoma are biologically the same or not is uncertain, further research based on the same standard will be necessary to reveal the pathophysiology of the undifferentiated carcinoma.

In the distal bile duct, 10 resected cases with undifferentiated features have been reported (Table 2) [7, 9, 10, 18–24]. Most tumors were sarcomatoid-type carcinoma, and there was no anaplastic-type carcinoma. Kajioka et al. previously reported spindle cell-type undifferentiated carcinoma in the distal bile duct and discussed the findings of a literature review [24]. Their cases also had early tumor recurrence 2 months after curative surgery. Furthermore, patients with undifferentiated tumors with lymph node metastasis and nerve invasion died within 1 year of surgery. The aggressive tumor biology of undifferentiated carcinoma in the distal bile duct was similar to perihilar undifferentiated carcinoma.

**Table 2:** Patient characteristics with undifferentiated carcinoma of the distal bile duct

Author	Sex/Age	Chief complaint	Gross appearance	Size (mm)	Surgical procedure	Resection status	Subtype	Prognosis (the cause of death)
Mokuno [18]	81/M	Jaundice	Nodular	92 × 33	PD	R0	Sarcomatoid	10 months dead (local recurrence)
Nagai [19]	78/M	General fatigue	Polypoid	10 × 10	PD	R0	Sarcomatoid	15 months alive
Yoon [9]	78/M	Jaundice Abdominal pain	Wall thickness	40 × 30	PD	R0	Sarcomatoid	POD5 dead (cardiac problem)
Kadono [10]	75/F	Jaundice	Wall thickness	–	PD	R0	Sarcomatoid	2 years dead (local recurrence)
Oikawa [20]	61/M	Jaundice Abdominal pain	Polypoid	48 × 27	PD	R0	Sarcomatoid	7 months alive
Fujikawa [21]	73/M	General malaise	Nodular	32 × 26	PD	R0	Not classifiable^*^	-
Kumei [22]	73/F	Jaundice Epigastralgia	Polypoid	65 × 35	PD	R0	Sarcomatoid	6 months dead (liver metastasis)
Zhang [7]	51/F	Jaundice Abdominal pain	Wall thickness	40 × 35	PD	R0	Carcinosarcoma	3 years alive
Sasamoto [23]	79/M	Jaundice	Nodular	18 × 13	PD	R0	Sarcomatoid	26 months dead (liver metastasis)
Kajioka [24]	76/M	Epigastric pain	Nodular	33 × 20	PD	R0	Sarcomatoid	65 days dead (peritoneal dissemination)

*PD* pancreatoduodenectomy

*The tumor cells resembled neuroendocrine or small cell carcinoma

To the best of our knowledge, this is the first case report of anaplastic-type perihilar undifferentiated carcinoma. Anaplastic-type carcinoma has frequently been reported in pancreatic cancer to exhibit rapid growth and have an unfavorable prognosis [25]. These characteristics are consistent with the present case.

Major hepatectomy with BDR is generally selected for perihilar cholangiocarcinoma. However, undifferentiated perihilar carcinoma was often treated by segmental BDR (*n* = 3). This may be because sarcomatoid tumors are often nodular in shape and well-circumscribed and BDR may more easily achieve a cancer-negative margin, in contrast to biliary adenocarcinoma, which often spreads widely along the bile duct. Previous case reports showed that segmental BDR for middle bile duct cancer resulted in less postoperative morbidity, but a worse prognosis than these major surgeries [26–28]. Therefore, BDR needs to be considered for patients with a poor general condition. Since the present case was older than 80 years, hemi-hepatectomy and BDR were considered to be highly invasive. Therefore, we initially planned segmental BDR with the aim of achieving cancer-negative surgical margins.

In terms of non-surgical treatment for perihilar undifferentiated carcinoma, two cases reportedly received systemic chemotherapy: adjuvant chemotherapy with gemcitabine and FOLFOX6 for advanced undifferentiated carcinoma at Klatskin-position [15, 29]. The former achieved tumor-free survival of 16 months after surgery, while the latter was in complete remission for 4 years. Two cases of perihilar undifferentiated carcinoma, including the present case, had very early recurrence after surgery. It is important not to miss an opportunity for systemic chemotherapy due to surgery.

## Conclusions

According to the literature search, this is the first case report of undifferentiated cholangiocarcinoma with anaplastic features. It is necessary to establish a category of anaplastic carcinoma in the classification of cholangiocarcinoma and clarify the diagnostic criteria for this rare subtype.

## Data Availability

Not applicable.

## Abbreviations

CT: Computed tomography
BDR: Bile duct resection
